# Distribution of Calcium, Phosphorus and Magnesium in Yak (*Bos grunniens*) Milk from the Qinghai Plateau in China

**DOI:** 10.3390/foods12071413

**Published:** 2023-03-27

**Authors:** Piero Franceschi, Wancheng Sun, Massimo Malacarne, Yihao Luo, Paolo Formaggioni, Francesca Martuzzi, Andrea Summer

**Affiliations:** 1 Department of Veterinary Science, University of Parma, Via del Taglio 10, 43126 Parma, Italy; 2 Animal Science Department, College of Animal Husbandry and Veterinary Medicine, Qinghai University, Nig Da Road 251, Xining 810016, China; 3 Department of Food and Drug, University of Parma, Parco Area delle Scienze, 27/A, 43124 Parma, Italy

**Keywords:** yak milk, protein fractions, mineral content, salt equilibria, casein micelles mineralisation

## Abstract

This research was aimed to assess the distribution of calcium, phosphorus and magnesium within the casein micelles of yak milk. To this aim, nine bulk yak milk samples (Y-milk), collected in three yak farms located in the Chinese province of Qinghai, were compared to nine bulk cow milk samples used as a reference. A quite similar content of colloidal calcium (0.80 vs. 0.77 mmol/g of casein; *p* > 0.05), a higher content of magnesium (0.05 vs. 0.04 mmol/g of casein; *p* ≤ 0.01) and a lower content of colloidal phosphorus (0.48 vs. 0.56 mmol/g of casein; *p* ≤ 0.01) between yak and cow casein micelles were found. Moreover, the yak casein micelles showed a lower value of prosthetic phosphorus (0.20 vs. 0.26 mmol/g of casein; *p* ≤ 0.05) compared to the cow micelles. The lower values of colloidal and prosthetic phosphorus in yak casein micelles suggest that the yak casein is less phosphorylated than the cow one.

## 1. Introduction

The heat stability of milk and its ability to transfer high quantities of Ca and P in a highly assimilable chemical form by the human organism depend on casein micelles structure. Moreover, the casein micelles are the substrate of the coagulation of milk (acid or enzymatic). This process is an essential step in milk cheesemaking and allows the efficient release of biological active components of milk during digestion. Casein micelles are organised roughly in spherical particles constituted by the four caseins and by an amorphous mineral cluster defined as colloidal calcium phosphate. In bovine milk, micellar minerals represent about 6% of the dry matter of casein micelles. Although there are several models of casein micelles structure, there is a general agreement about the stabilising effect played by k-casein on the surface of the micelles and by the nanoclusters of calcium phosphate in the internal zones of the micelles. A quantitative model of the bovine milk casein micelle, characterized by ion equilibrium and calcium phosphate sequestration by individual caseins, was recently proposed by Bijl et al. [1].

The degree of mineralisation (or mineralisation level) of micelles can be defined as the concentration of Ca, Mg and P within the casein micelle, in the form of calcium phosphate nanoclusters or via ionic bonds with amino acid residues [1,2]. The degree of mineralisation of casein micelles influences the processing and nutritional properties of milk and of the products derived from it. In particular, a high level of mineralisation of casein improves the rennet coagulation ability of milk but seems to decrease the degradation of casein during in vitro gastric digestion [3,4].

Studies on the degree of mineralisation of casein micelles mainly concerned bovine milk casein, although milk from other species was also examined. For example, in the last years, some studies investigated the degree of mineralisation of casein in milk from sheep [5,6], goat [6,7], buffalo [7,8,9], camel [10], donkey [11,12,13] and wild ruminants [14] such as red deer (*Cervus elaphus*), fallow deer (*Dama dama*), roe deer (*Capreulus capreulus*).

Yaks are extensively raised in the plateau of the western Tibetan region of China at altitudes ranging approximately from 2000 to 5000 m above the sea level [15,16]. Generally, yaks are raised mainly for their milk, their meat and their wool, which are a vital part of the local economy in the Tibetan region of China [16]. In particular, yak milk is a food product of great value for the population of the Qinghai plateau, where yaks are the only raised animals producing milk [15]. Indeed, yak milk, besides being a beverage, is used to produce many products. The main product is butter, but this milk is used for a variety of other products as well, such as yogurt and fermented beverages, hard and soft cheeses and other traditional products [17].

Nowadays, approximately 25% of yak milk is processed at industrial level [18] and, for its relevance in the nutrition of the people living in the Tibetan plateau, during the last years, several studies were carried out aimed to characterise the milk yield, chemical composition, cheesemaking aptitude and nutritional properties.

Yak average daily milk yield ranges from about 0.8 to 3.2 kg/d, according to the breed and rearing zone. Furthermore, also the length of lactation is variable, depending on the same factors, from 100 to 180 days, and this leads to an average milk production from 150 to 500 kg/lactation [16].

Compared to cow milk, yak milk has a higher concentration of milk constituents such as fat, protein and casein [15,19,20,21], a larger casein micelles size, better rennet coagulation properties and a higher cheese yield [22,23]. Moreover, it was observed that yak casein is less soluble than the cow one [24] and that these proteins differ from each other in composition and hydration [25].

However, to date, only a scarce number of studies were carried out to analyse yak milk mineral composition. Yak milk has a higher content of Ca, P and Mg than cow milk [26,27,28]. Most of Ca and P are in the colloidal phase, whereas 3/4 of Mg is in the soluble phase [27]. Nowadays, no one has yet investigated casein micelles mineralisation in yak milk in its native state.

For this reason, the knowledge of the characteristics of casein micelles and their mineral content can be useful to exploit the dairy potential and address the transformation technology of yak milk for its valorisation. Thus, the characterisation of milk from yak was carried out in this study, focusing on its mineral content and the distribution of Ca, P and Mg between its soluble and micellar phases, as well as their concentration within casein micelles.

## 2. Materials and Methods

### 2.1. Experimental Design and Sampling Procedure

Nine bulk yak milk samples were collected in three yak farms located in three different zones of the Chinese province of Qinghai. All animals belonged to Plateau yak breed. One herd was from the province of Guoluo (3719 m a.s.l.), one herd from the province of Hainan (2835 m a.s.l.), and one herd from the province of Wulan (2960 m a.s.l.). Samples were collected monthly from June to August in each herd. For the purpose of comparison, nine bulk milk samples of Italian Friesian cows were collected and analysed with the same method. These latter were taken from three farms located in the north of Italy raising only Italian Friesian cows. As for the yak milk samples, the Italian Friesian milk samples were collected monthly from June to August in each herd.

The milk samples were representative of the herd bulk milk and were collected at the end of the morning milking. After sampling, they were frozen, transported to the laboratories and kept at −20 °C until the analysis.

### 2.2. Analytical Methods

For each milk sample, total N (TN), non-casein N (SN) and non-protein N (NPN) were determined by the Kjeldahl method [29,30,31] in milk, milk acid whey at pH 4.6 and trichloroacetic acid filtered whey (TCA 120 g/L; Carlo Erba Reagents, I-20010, Milan, Italy), respectively. The Kjeldahl method was performed using a DK6 Digestor and an UDK126A Distiller (VELP Scientifica, Usmate, Italy).

Moreover, for each milk sample, also by the Kjeldahl method, not-coagulable N (NCN) on the rennet whey was determined. The rennet whey was obtained from milk, according to Franceschi et al. [32], adding 2 mL of diluted 1:100 rennet (Christian Hansen, DK-7172 Hørsholm, Denmark) into 100 mL of milk previously thermostated at 35 °C. After coagulation of the milk, the whey outcome by curd syneresis was filtered on Whatman 1 paper filter (Merck Millipore Corporation, D-64293, Darmstadt, Germany).

From these data, crude protein (TN × 6.38/1000), whey protein (SN × 6.38/1000), casein ((TN-SN) × 6.38/1000), casein number ((TN-SN) × 100/TN) and NPN × 6.38 (NPN × 6.38/1000) were calculated as described by Franceschi et al. [33], and paracasein was calculated ((TN-NCN) × 6.38/1000) according to Franceschi et al. [34].

The lactose and fat contents were determined by the mid-infrared method [35] using a MilkoScan FT 6000 (Foss Electric, DK-3400, Hillerød, Denmark). Moreover, somatic cells and total bacterial count were assessed using the fluoro-opto-electronic method [36] with Fossomatic (Foss Electric, DK-3400, Hillerød, Denmark) and the flow cytometry method [37] with BactoScan FC (Foss Electric, Hillerød, Denmark), respectively, and dry matter (DM) was obtained after oven-drying 20 g of milk at 102 °C [38].

Furthermore, each milk sample was skimmed and subsequently submitted to ultrafiltration process in Amicon 8200 ultrafiltration cells (Merck Millipore Corporation, Darmstadt, Germany). The ultrafiltration process was performed as described by Petrera et al. [39] with a Millipore membrane with 30 kDa cut-off in a N_2_ flow at 75 psi (polyethersulfone ultrafiltration membranes, Merck Millipore Corporation, Darmstadt, Germany).

The ash content was obtained by muffle calcination at 530 °C of 20 g of milk and of 10 g of ultrafiltered whey [40].

The ashes were solubilised in hydrochloric acid to obtain a hydrochloric ash solution [41] and, from this, by a colorimetric method [42], total P and soluble P were assessed in the hydrochloric ash solution of milk and in the hydrochloric ash solution of ultrafiltered whey, respectively. Moreover, also by the colorimetric method of Allen [42], the content of total acid-soluble P was assessed in trichloroacetic (TCA) acid-filtered whey digested at 240 °C by a DK6 digestion unit (VELP Scientifica, Usmate, Italy) for 1 h in perchloric acid 65% (Carlo Erba Reagents, I-20010, Milan, Italy). The colorimetric method of Allen, in brief, was performed by adding to 10 mL of hydrochloric ash solution, diluted 40 times, 2 mL of perchloric acid 65%, 2 mL of a solution with 20 g/L of 2,4-diaminophenol dihydrochloride, 200 g/L of sodium metabisulfite and 1 mL of ammonium molybdate (83 g/L) solution (all reagents came from Carlo Erba Reagents, I-20010, Milan, Italy). After 25 min, 1 mL of this solution was read by a Helios spectrophotometer (Thermo Fisher Scientific, Waltham, MA 02451, USA) at 750 nm. For the determination of P 5, standard solutions from 25 mg/100 g to 400 mg/100 g were used (KH_2_PO_4_, Carlo Erba Reagents, I-20010, Milan, Italy).

Moreover, starting from the hydrochloric ash solution of the milk and of the ultrafiltered whey, opportunely diluted ten thousand times, the total content of Ca and Mg and their content in the solution were determined [41] by atomic absorption spectrometry using a Perkin-Elmer 1100 B instrument (Perkin-Elmer, Waltham, MA 02451, USA). For both Ca and Mg determination, calibration curves were obtained using 5 standard solutions, (CaCl_2_·6H_2_O and MgCl_2_·6H_2_O of Carlo Erba Reagents, I-20010, Milan, Italy) ranging from 0.5 to 8 ppm for Ca determination and ranging from 0.05 to 0.8 ppm for Mg determination.

From these data, the colloidal fractions, namely, the minerals inside the casein micelles, of Ca and Mg were calculated as the difference between their total and soluble contents. Differently from Ca and Mg, colloidal P, within casein micelles, is present in two different chemical forms, i.e., as part of the phosphorylated residues of caseins (casein P) and as a constituent of colloidal inorganic P. These fractions were calculated according to Malacarne et al. [12] as follows:

Colloidal P = TP − SP(1)

Casein P = TP − TASP(2)

Colloidal inorganic P = TASP − SP(3)

where TP = total phosphorus; SP = soluble phosphorus; TASP = total acid-soluble phosphorus.

Furthermore, colloidal P content was corrected for the quota of P in phospholipids according to Bonaga and Mascolo [43].

Then, the ratios of the mineral soluble contents with respect to their total amounts were calculated and, according to Malacarne et al. [12], as well as the ratios between colloidal minerals and casein, expressed in millimoles per gram of casein, were calculated.

Finally, the pH was measured by a Crison potentiometer (Crison Instruments, E-08328, Barcelona, Spain), and the density at 15 °C by means of a Quevenne lactometer.

### 2.3. Statistical Analysis

The data collected were tested by analysis of variance, using the general linear model procedure of SPSS (IBM SPSS Statistics 26, Armonk, NY 10504-1722, USA), according to the following hierarchical model:

Y_ijkl_ = µ + S_i_ + T_j_ + H_ik_ + ε_ijkl_ (4)

where Y_ijkl_ = dependent variable; µ = overall mean; S_i_ = effect of the species (i = 1, 2); T_j_ = effect of the trial (j = 1, …, 3); H_ik_ = effect of herd nested within species (1, …, 5); ε_ijkl_ = residual error.

The significance of the differences between yak milk and cow milk was tested by the Bonferroni post-hoc test.

## 3. Results

In Table 1, the chemical composition, physicochemical properties and counts of somatic cells and total bacteria of yak bulk milk (Y-milk) and cow bulk milk (C-milk) are shown.

The average values of dry matter, crude protein, whey protein, casein, NPN × 6.38 true protein, true whey protein and paracasein were different in Y-milk and C-milk, with *p* ≤ 0.001. Furthermore, Y-milk and C-milk showed different average values of fat, somatic cells count and total bacterial count, with *p* ≤ 0.01, and of lactose and ash, with *p* ≤ 0.05.

The content of dry matter and its main constituents (lactose, fat, crude protein and ash), as well as of crude protein and its fractions (whey protein, casein, paracasein and NPN × 6.38) and the total bacterial count were higher in Y-milk than in C-milk. On the contrary, the somatic cell count was found to be lower in Y-milk than in C-milk. The casein number and the values of density and pH were not different between the two types of milk.

In Table 2, the contents of Ca, P and Mg and their distribution between the colloidal and the soluble phases of Y-milk and C-milk are reported.

Among the considered minerals, both in Y-milk and in C-milk, Ca was the most abundant mineral, P being the second, and Mg the third.

The total contents of Ca, P and Mg and their fractions as well, except for casein P that was not significantly different, were higher in Y-milk than in C-milk.

On the other hand, the percentage ratios of soluble Ca and soluble Mg with respect to their total contents were lower in Y-milk than in C-milk, whereas no significant differences were observed for the percentage ratio of soluble P with respect to total P.

In Table 3, the concentrations of Ca, P and Mg within the casein micelles of Y-milk and C-milk, expressed as mmol per gram of casein, are reported.

The contents of colloidal Ca and colloidal Mg and the colloidal-Ca-to-colloidal-P ratio showed significant differences between Y-milk and C-milk (*p* ≤ 0.01), while casein P contents showed a significant difference between Y-milk and C-milk, with *p* ≤ 0.05.

The casein micelle of Y-milk had lower content of P and higher content of Mg, than C-milk. Finally, no differences between the two types of micelles were observed for Ca.

## 4. Discussion

### 4.1. Yak Milk Chemical Composition and Physico-Chemical Properties

In general, the results of the main studies concerning the chemical composition of yak milk were reported in several comparative reviews [44,45,46,47,48]. The concentration of the main constituents of Y-milk (ash, lactose, fat and crude protein) were within the ranges reported by previous studies [15,16,49]. Differently from C-milk, in which the principal constituent of dry matter was lactose, in Y-milk the main constituent of dry matter was fat (33.22 g 100 g^−1^ of dry matter). In addition, the values of the protein fractions were comparable with those reported in previous studies [18,20,49]. The higher casein content of Y-milk, compared to C-milk, resulted in a higher paracasein content, which is the rennet-coagulable fraction of casein.

Nevertheless, since the cheesemaking process consists in the formation of a three-dimensional network of paracasein, in which fat globules and part of the whey are entrapped [50], milk the casein and paracasein contents are very important traits, for their repercussions on the yield of both soft cheeses [51,52] and hard cheeses [53,54]. Indeed, the cheesemaking yield is directly proportional to the milk casein and paracasein contents for both soft and hard cheese production, as reported by many authors [23,33,51,52,53,54]

From this point of view, the yak milk high contents of casein, paracasein and fat can result in a high cheese-yielding ability, as demonstrated by Zhang et al. [23]. The casein number of Y-milk in the present research was higher than that reported by Li et al. [15], who found an average value of 74.63% from 104 individual milk samples collected from Maiwa breed yaks. This difference may depend on genetic differences between Maiwa and Plateau yak breeds.

The Y-milk somatic cells average content was slightly higher than 100,000 cells/mL of milk. Currently, there is not a clear threshold limit to assess intra-mammary infections (IMI) in yak. If we consider the threshold limit for somatic cells commonly accepted for individual cows reared in an intensive system (200,000 cells/mL), the value in yak bulk milk observed seemed to indicate a low prevalence of IMI in the yak herds sampled here. Moreover, it is important to highlight that a high somatic cells content in cow milk has negative effects on its rennet coagulation aptitude [55,56,57] and, consequentially on the cheese-making efficiency [50] and milk cheese-yielding ability [33,58].

In contrast, the total bacterial count in Y-milk was very high when compared to that in C-milk one. This was probably due to differences between the raising systems of the yaks and cows involved in this research. Indeed, C-milk was collected from a free-stall herd, the more widespread housing system in Italy, and milking was mechanised and carried out in a milking parlor [59]. With this system, the collected milk is transported through pipes to a refrigeration tank where it is cooled. The limited contact between the milk and the environment and the cooling of the milk keeps the total bacterial count low [60], and refrigeration contributes to reducing the bacterial growth [61]. On the opposite, yaks were raised in high mountain pastures and were not reared in a stall, the milking procedure was manually performed, and the milk was not immediately cooled after milking, conditions that can all promote bacterial growth.

Clearly, improving the hygiene of the milking practices and cooling the yak milk at the farm could be the best method to reduce somatic cell count and bacterial growth and to limit the proteolytic and lipolytic activity of enzymes that can alter the milk quality [62].

Finally, the average pH value was consistent with that reported by Zhang et al. [63] in a study on the factors influencing the rennet-induced coagulation properties of yak milk.

### 4.2. Yak Milk Mineral Content, Salt Equilibrium and Casein Micelles Mineralisation

Cui et al. [28], in a study carried out on milk from the same yak breed considered here, found mineral contents quite higher than those observed in this study, using a different method to assess mineral concentration (inductively coupled plasma atomic emission spectroscopy), i.e., 227, 170 and 14.5 mg/100 g of milk for Ca, P and Mg, respectively. The contents of total Ca and P were slightly higher, and that of Mg slightly lower than those showed by Li et al. [15], reporting for Maiwa Y-milk average values of 1545.45, 922.04 and 154.10 mg/kg of milk for Ca, P and Mg, respectively.

Moreover, the percentages of soluble Ca and P were lower and higher, respectively, than those reported by Wang et al. [27] for Y-milk produced by yak raised in the Qinghai-Tibetan Plateau.

The higher concentrations of Ca, P and Mg in Y-milk compared to C-milk depended mainly on the high amount of casein in the former milk with respect to the latter one. Indeed, Ca, P and Mg contribute to the casein micelle structure, and thus, the milk casein content positively affects the colloidal contents of Ca, P and Mg [64].

In general, the data om micelle mineralisation in C-milk are in agreement with the results of previous studies carried out on individual and bulk milk samples [2,3]. Actually, colloidal P is composed of two different fractions: inorganic P, which represents P in inorganic calcium phosphate within the casein micelles, and casein P, corresponding to phosphorus in the phosphorylated amino acid residues of caseins [3].

Differences were observed for inorganic P, while Y-milk showed a lower content of casein P than C-milk. Thus, the micelles of the two types of milk had the same concentration of inorganic salts, and Y-milk had a lower number of phosphorylated amino acid residues. This observation was confirmed by the values of the colloidal-Ca-to-colloidal-P ratio and of the colloidal-Ca-to-colloidal-inorganic-P ratio. Indeed, the first value was higher in Y-milk than in C-milk, while the second did not show a significant difference when comparing the milk of the two species.

## 5. Conclusions

Yak milk appeared to be characterised by a high content of casein and, therefore, of minerals that contribute to the casein micelle structure. In particular, the milk of yak seems to be extremely rich in Ca, P and Mg, especially in their colloidal forms, which should positively influence the bioavailability of Ca and P during the digestion process.

Finally, in yak milk, the lower casein P content per casein unit suggests that the yak caseins are less phosphorylated than the cow ones and have a lower number of phosphorylated amino acid residues within the casein micelles. This feature may affect the casein micelle structure, with repercussion on the processability and digestion of yak milk casein when compared to the cow one.

However, it should be considered that this study was conducted on bulk milk samples, using milk from only one breed (Plateau yak breeds) of yak.

Therefore, it could be important, in the future, to expand this research, examining milk from other yak breeds.

## Figures and Tables

**Table 1:** Least-square means of chemical composition, physico-chemical properties and counts of somatic cells and total bacteria for yak (*Bos grunniens*, Y-milk) and cow (*Bos taurus*, C-milk) milk.

Parameters	Unit of Measure	Y-Milk n ^1^ = 9	C-Milk n ^1^ = 9	SE ^2^	*p* ^3^
Dry matter	g/100 g	18.57	12.25	0.98	***
Ash	g/100 g	0.78	0.71	0.03	*
Lactose	g/100 g	4.79	4.95	0.04	*
Fat	g/100 g	6.17	3.46	0.34	**
Crude protein	g/100 g	4.57	3.16	0.10	***
Whey protein	g/100 g	1.05	0.71	0.02	***
Casein	g/100 g	3.53	2.45	0.07	***
Casein number	%	77.12	77.58	0.17	NS
NPN × 6.38	g/100 g	0.26	0.17	0.01	***
True protein	g/100 g	4.31	2.99	0.10	***
True whey protein	g/100 g	0.79	0.54	0.02	***
Paracasein	g/100 g	3.05	2.87	0.08	***
Density	Kg/L	1.034	1.032	0.01	NS
pH-value	value	6.69	6.70	0.02	NS
Somatic cells count	10^3^ cells/mL	101.69	311.88	29.14	**
Total bacterial count	10^3^ FCU/mL	890.99	82.11	285.25	**

^1^ Number of samples; ^2^ Standard error; ^3^ *p*-value: NS *p* > 0.05; * *p* ≤ 0.05; ** *p* ≤ 0.01; *** *p* ≤ 0.001.

**Table 2:** Least-square means of Ca, P and Mg contents and distribution between the colloidal and the soluble phases of yak (*Bos grunniens*, Y-milk) and cow (*Bos taurus*, C-milk) milk.

Parameters	Unit of Measure	Y-Milk n ^1^ = 9	C-Milk n ^1^ = 9	SE ^2^	*p* ^3^
Ash of ultrafiltered whey	g/100 g	0.56	0.54	0.02	NS
Total Ca	mg/100 g	160.74	113.65	7.25	**
Colloidal Ca	mg/100 g	112.62	76.01	6.21	**
Soluble Ca	mg/100 g	48.12	37.64	1.33	***
Total P	mg/100 g	113.88	87.70	3.91	**
Colloidal P	mg/100 g	51.96	42.39	1.68	**
Colloidal inorganic P	mg/100 g	30.27	22.34	1.15	**
Casein P	mg/100 g	21.70	20.05	1.70	NS
Soluble P	mg/100 g	58.40	43.20	3.06	**
Total Mg	mg/100 g	12.52	9.49	0.51	**
Colloidal Mg	mg/100 g	4.40	2.28	0.20	***
Soluble Mg	mg/100 g	8.12	7.22	0.33	*
Soluble Ca to total Ca ratio	g/100 g of total Ca	30.24	33.13	0.87	*
Soluble P to total P ratio	g/100 g of total P	50.70	49.24	1.03	NS
Soluble Mg to total Mg ratio	g/100 g of total Mg	64.88	76.06	0.52	***

^1^ Number of samples; ^2^ Standard error; ^3^ *p*-value: NS *p* > 0.05; * *p* ≤ 0.05; ** *p* ≤ 0.01; *** *p* ≤ 0.001.

**Table 3:** Least-square means of Ca, P and Mg concentrations within the casein micelles of yak (*Bos grunniens*, Y-milk) and cow (*Bos taurus*, C-milk) milk.

Parameters	Unit of Measure	Y-Milk n ^1^ = 9	C-Milk n ^1^ = 9	SE ^2^	*p* ^3^
Casein	g 100 g^−1^	3.53	2.45	0.07	***
Colloidal Ca	mmol/g of casein	0.80	0.77	0.05	NS
Colloidal P	mmol/g of casein	0.48	0.56	0.02	**
Colloidal inorganic P	mmol/g of casein	0.28	0.29	0.01	NS
Casein P	mmol/g of casein	0.20	0.26	0.02	*
Colloidal Mg	mmol/g of casein	0.05	0.04	0.01	**
Colloidal Ca to colloidal P	value	1.67	1.39	0.05	**
Colloidal Ca to colloidal inorganic P	value	2.95	2.66	0.24	NS
Soluble Ca to soluble P	value	0.67	0.68	0.02	NS

^1^ Number of samples; ^2^ Standard error; ^3^ *p*-value: NS *p* > 0.05; * *p* ≤ 0.05; ** *p* ≤ 0.01; *** *p* ≤ 0.001.

## Data Availability

The data presented in this study are available on request from the corresponding author.
